# Differentiation of Schizophrenia Patients from Healthy Subjects by Mismatch Negativity and Neuropsychological Tests

**DOI:** 10.1371/journal.pone.0034454

**Published:** 2012-04-05

**Authors:** Yi-Ting Lin, Chih-Min Liu, Ming-Jang Chiu, Chen-Chung Liu, Yi-Ling Chien, Tzung-Jeng Hwang, Fu-Shan Jaw, Jia-Chi Shan, Ming H. Hsieh, Hai-Gwo Hwu

**Affiliations:** 1 Department of Psychiatry, National Taiwan University Hospital Yun-Lin Branch, Dou-Liou City, Yun-Lin, Taiwan; 2 Department of Psychiatry, National Taiwan University Hospital and College of Medicine, National Taiwan University, Taipei, Taiwan; 3 Neurobiology and Cognitive Science Center, National Taiwan University, Taipei, Taiwan; 4 Department of Neurology, National Taiwan University Hospital, Taipei, Taiwan; 5 Institute of Biomedical Engineering, National Taiwan University, Taipei, Taiwan; 6 Department of Psychiatry, Cathay General Hospital, Taipei, Taiwan; Chiba University Center for Forensic Mental Health, Japan

## Abstract

**Background:**

Schizophrenia is a heterogeneous disorder with diverse presentations. The current and the proposed DSM-V diagnostic system remains phenomenologically based, despite the fact that several neurobiological and neuropsychological markers have been identified. A multivariate approach has better diagnostic utility than a single marker method. In this study, the mismatch negativity (MMN) deficit of schizophrenia was first replicated in a Han Chinese population, and then the MMN was combined with several neuropsychological measurements to differentiate schizophrenia patients from healthy subjects.

**Methodology/Principal Findings:**

120 schizophrenia patients and 76 healthy controls were recruited. Each subject received examinations for duration MMN, Continuous Performance Test, Wisconsin Card Sorting Test, and Wechsler Adult Intelligence Scale Third Edition (WAIS-III). The MMN was compared between cases and controls, and important covariates were investigated. Schizophrenia patients had significantly reduced MMN amplitudes, and MMN decreased with increasing age in both patient and control groups. None of the neuropsychological indices correlated with MMN. Predictive multivariate logistic regression models using the MMN and neuropsychological measurements as predictors were developed. Four predictors, including MMN at electrode FCz and three scores from the WAIS-III (Arithmetic, Block Design, and Performance IQ) were retained in the final predictive model. The model performed well in differentiating patients from healthy subjects (percentage of concordant pairs: 90.5%).

**Conclusions/Significance:**

MMN deficits were found in Han Chinese schizophrenia patients. The multivariate approach combining biomarkers from different modalities such as electrophysiology and neuropsychology had a better diagnostic utility.

## Introduction

Schizophrenia is recognized as a neurobiological syndrome with heterogeneous presentation and pathophysiology. The development of biological markers is important in schizophrenia research, which is restricted by the phenomenology-based diagnostic system. Biological markers are measurable traits that are specific to particular conditions and have diagnostic and predictive values. Several measurements have been reported to discriminate schizophrenia patients from healthy controls, such as quantitative electroencephalography and event-related potentials (ERP) [1]. However, a single marker may not be able to address the heterogeneous nature of schizophrenia.

Mismatch negativity (MMN) is a negative component of auditory event-related potentials elicited when infrequent discernible deviant sounds (“oddballs”) occur in a sequence of repetitive standard sounds. The MMN response relies on the established memory trace of standard sounds and is an index of automatic pre-attentive sensory processing of auditory input and echoic memory [2]–[6]. MMN deficit has been shown to be a robust feature for chronic schizophrenia patients [7] and is regarded as a candidate endophenotype for schizophrenia [8], [9]. Some studies suggested that MMN deficit is specific to schizophrenia [10]–[14] and is unrelated to neuroleptics treatment [15]–[19]. However, MMN deficit has ever been observed for subjects with bipolar disorder [20]–[21] or Asperger syndrome [22]–[24]. Furthermore, MMN alone may not be adequate to predict whether an individual subject has schizophrenia or not. The effect size of MMN deficit for schizophrenia is around 0.99, and it implies that the distributions of MMN of controls and schizophrenia patients overlap [7]. In the literature, only one study has applied MMN in the context of multivariate electrophysiological endophenotype approach (MMN, P50, P300, and antisaccades) to predict the diagnostic groups [25]. The study found that a weighted combination of the four markers could provide better power in prediction. In addition to the multivariate approach, using markers measured by different modalities may improve the predictive power further [26]. To date, there have been no study on MMN in Han Chinese schizophrenia patients. The current study thus aims to evaluate the performance of combining MMN with neuropsychological tests to differentiate schizophrenia patients from healthy subjects in a population of Han Chinese Ethnicity.

The pattern of discriminating schizophrenia patients from healthy subjects by MMN is related to the types of deviant stimuli and aging. Todd et al. showed that patients at the early course of schizophrenia had deficits in duration and intensity MMN, but not frequency MMN. With longer length of illness, the frequency MMN deficit became significant [27]. Frequency MMN deficits were not found in first-episode schizophrenia patients [28]–[30] or patients with recent-onset schizophrenia [31]. One study found significant reduction in chronic schizophrenia patients and marginal reduction in recent-onset schizophrenia patients for both duration and frequency MMN [31]. Marginally decreased duration MMN amplitudes, rather than frequency MMN was noted for subjects exhibiting prodromal symptoms of schizophrenia [32]. These studies suggested MMN deficits are related to the progression of schizophrenia, and duration MMN might be a more sensitive marker in the early stage of the disease. However, two studies were unable to find duration MMN deficits in first-episode schizophrenia patients [29], [30]. In addition, the age-related declination of MMN observed in healthy subjects further complicated the interpretation of progressive MMN reduction with the course of schizophrenia [33]–[38]. Salisbury et al. conducted a follow-up study for a group of first-hospitalized schizophrenia patients. Frequency MMN amplitude of schizophrenia patients was not different from age-matched controls initially, but 1.5 years later patients showed significant MMN reduction which was correlated with the reduction of left Heschl's gyrus gray matter [39]. The study by Todd et al. found significant age-related decline of duration MMN for both healthy subjects and schizophrenia, but the age-related decline of frequency MMN was only observed in schizophrenia patients [27]. Similarly, Kiang et al. also found age-related decline of duration MMN for both controls and schizophrenia patients [40]. In summary, with the course of schizophrenia, the duration MMN deficit remains stable and its gradual reduction seems to be related to aging. To the contrary, frequency MMN is related more to the progression of pathology of schizophrenia. Therefore, we chose to look at duration MMN for the prediction of diagnostic groups.

Cognitive impairment is a core feature of schizophrenia, with high intra-group heterogeneity [41]. The effect size of a single neuropsychological test to differentiate patients from healthy controls has been reported to be around 0.46 to 1.57, with the largest effects in global verbal memory and processing speed measured by digit symbol coding [42], [43]. But the distribution of test scores of schizophrenia patients overlapped with that of healthy subjects, and no single test was able to satisfactorily separate the two groups. The Continuous Performance Test (CPT) and Wisconsin Card Sorting Test (WCST) are two neuropsychological tests widely applied in the research of schizophrenia. The CPT is an index of sustained attention, early visual information processing, and response inhibition. During the CPT session, numbers from 0 to 9 are randomly presented to the subjects. They need to respond whenever the number “9” appears following the number “1”. The WCST is used as a complex measurement of executive function, where mental flexibility, working memory, and goal-directed behaviors are involved. The WCST requires subjects to match a series of 128 response cards one by one to the stimulus cards according to color, form, or number. After each trial, “right” or “wrong” is fed-back without telling the correct sorting principle. Subjects have to figure out the right principle and correctly complete 10 consecutive trials. Then the sorting rule changes, and subjects need to find the new rule. Schizophrenia patients have poorer performance on both the CPT and WCST, with reported effect sizes of 0.66 to 1.13 and 0.81 to 1.00 respectively [42].

In this study, we applied a multivariate approach to classify schizophrenia patients and healthy subjects using MMN and several neuropsychological markers (CPT, WCST, and the intelligence test). We first explored the MMN deficit and potential covariates. On developing the predictive model, the correlation between the two groups of markers were analyzed. Then in logistic regression modeling, useful predictive markers were selected and combined to construct a probability model to predict diagnostic grouping.

## Results

### Subjects

Comparisons of demographics and neuropsychological tests between the schizophrenia patients and healthy subjects are shown in Table 1. The mean Positive And Negative Syndrome Scale (PANSS) score of the patients was 53.0±15.1. Twenty percent of them received first generation antipsychotics, 62.5% received second generation antipsychotics, and the remaining 17.5% received clozapine. The mean chlorpromazine equivalent dose was 379.8±243.9 mg/day.

**Table 1:** Demographic data and clinical correlates.

Characteristics	Control	Schizophrenia	*P* value
Female – no. (%)	46 (60.5)	62 (51.7)	0.241
Age – years	36.25±1.12	37.96±9.83	0.264
Education – years	15.73±3.52	13.08±2.84	<0.001*
Smoking – PPD	0.048±0.20	0.17±0.41	0.016*
**CPT**			
d′	−0.03±1.06	−0.68±1.20	<0.001*
md′	−0.08±0.99	−0.90±1.32	<0.001*
**WCST**			
Perseverative errors	−0.13±0.95	0.78±1.43	<0.001*
Categories achieved	0.37±1.05	−0.50±1.04	<0.001*
Trials to complete first category	0.04±0.91	0.47±1.19	0.010*
Conceptual level response	0.26±1.05	−0.62±1.15	<0.001*
**WAIS-III**			
Arithmetic	11.96±3.15	8.22±3.23	<0.001*
Digit Span	12.38±3.14	9.76±4.15	<0.001*
Information	11.99±2.92	10.07±3.23	<0.001*
Digit Symbol-coding	11.90±2.92	10.46±12.81	0.350
Block Design	11.86±2.88	10.03±6.87	0.034*
Working Memory Index	112.14±15.30	92.10±17.57	<0.001*
Verbal IQ	112.67±16.22	94.53±17.08	<0.001*
Performance IQ	113.06±16.56	90.61±16.84	<0.001*
Full Scale IQ	112.25±18.88	92.52±15.63	<0.001*

* Significant difference between controls and patients with schizophrenia (significant level at 0.05)Independent *t* test for continuous variables.

Pearson's chi-square (2-sided) test for categorical variables.

PPD: package per day.

d′: sensitivity index of undegraded CPT.

md′: sensitivity index of degraded CPT.

### Mismatch negativity

**Figure 1:**
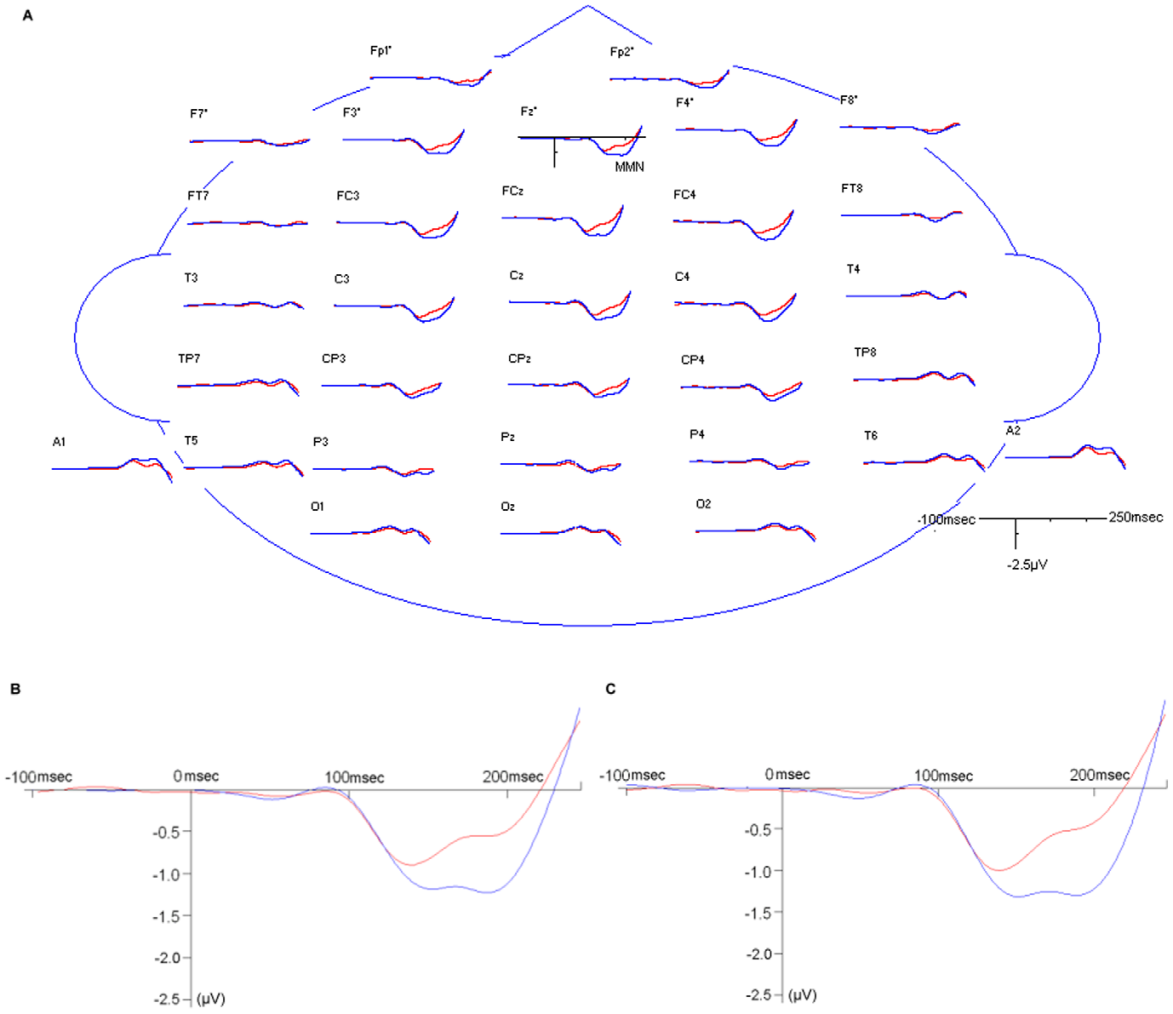
Grand average mismatch negativity waveforms. (**A**) Grand average mismatch negativity waveform at each electrode shown for schizophrenia patients (red line) and healthy subjects (blue line). The mismatch negativity waveform reversed in polarity at the mastoid electrodes. (**B**) Grand average MMN waveform at electrode Fz. (**C**) Grand average MMN waveform at electrode FCz.

**Figure 2:**
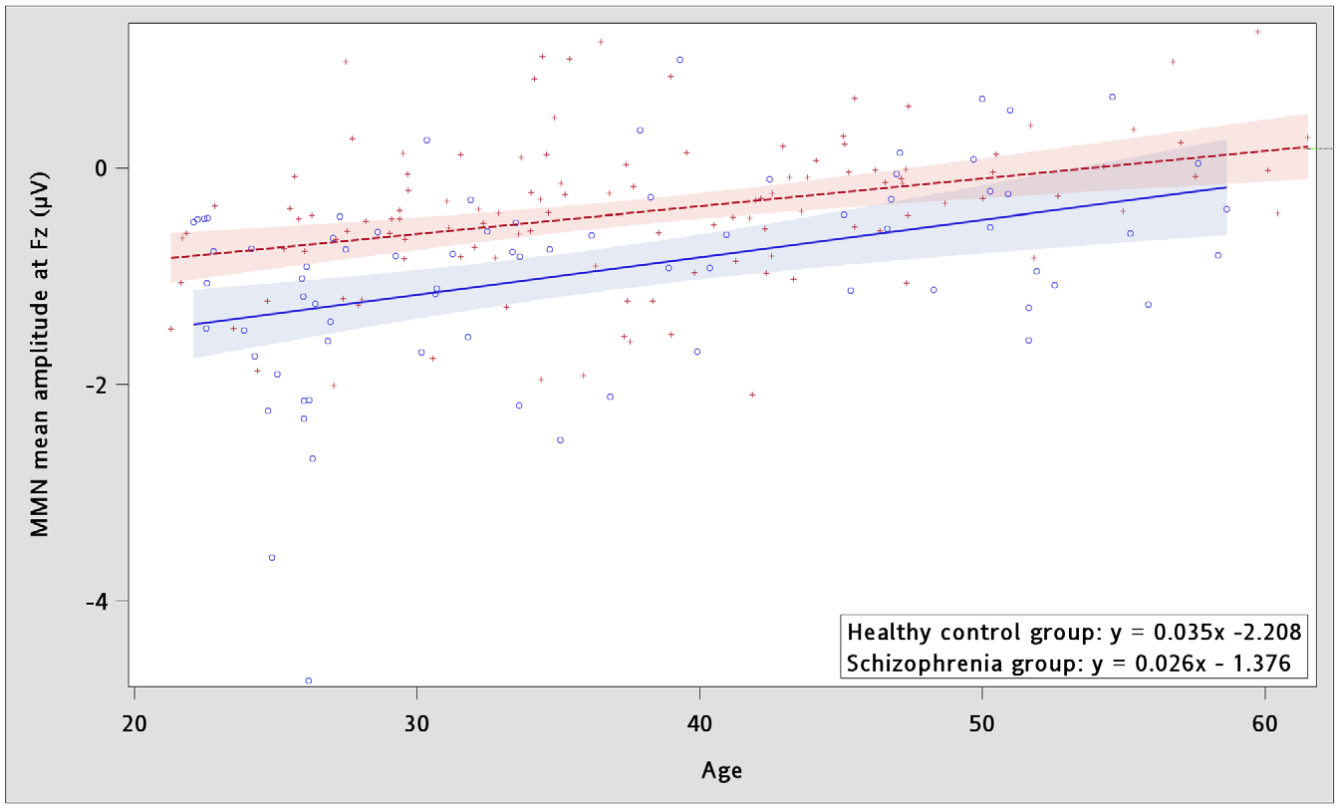
Effects of age on mismatch negativity. Mean mismatch negativity amplitude (135–205 msec) at electrode Fz reduces with aging. The reduction rate were not significantly different between healthy control group (open circles and solid regression line) and schizophrenia group (crosses and dashed regression line).

Grand average MMN waveforms for each group are shown in Figure 1. Repeated-measures ANOVA showed a significant main effect of group (F(1,182) = 6.57, *p* = 0.0112) and electrode x group interaction (F(31,5642) = 11.78, *p*<0.0001, Huynh-Feldt ε = 0.1268) on MMN across electrodes. Age was a significant covariate (age main effect: F(1,182) = 10.41, *p* = 0.0015; age x electrode: F(31,5642) = 13.85, *p*<0.0001, ε = 0.1268) and was thus included in following analyses. In both schizophrenia and control groups, amplitudes of MMN reduced with increasing age, and the regression coefficients were not different between groups (t = −0.89, df = 1, *p* = 0.3746) (Figure 2). Duration of illness was not a significant covariate after controlling for the effect of age. The MMN amplitudes at individual electrodes and their effect sizes are shown in Table 2. The largest effect size was seen at electrode FCz. The midline analysis showed more negative MMN frontally in both groups (electrode main effect: F(5,950) = 53.41, *p*<0.0001, ε = 0.3245). MMN peaked at FCz in control group and at Fz in schizophrenia group. MMN at frontal electrodes was not correlated with any neuropsychological tests or PANSS scores.

**Table 2:** Comparison of mismatch negativity amplitudes between groups.

	Control	Schizophrenia	t value	*P* value	Effect size
Fp1	−0.45±0.51	−0.2±0.39	3.89	<0.001*	0.55
Fp2	−0.48±0.57	−0.21±0.47	3.63	<0.001*	0.52
F7	−0.3±0.48	−0.16±0.41	2.16	0.032	0.31
F8	−0.23±0.76	−0.09±0.63	1.4	0.163	0.21
Fz	−0.96±0.94	−0.41±0.69	4.71	<0.001*	0.66
F3	−0.82±0.86	−0.34±0.63	4.49	<0.001*	0.63
F4	−0.9±0.88	−0.39±0.64	4.76	<0.001*	0.66
FT7	−0.18±0.54	−0.09±0.45	1.24	0.216	0.18
FT8	−0.12±0.74	−0.07±0.48	0.59	0.559	0.09
FC3	−0.87±0.90	−0.36±0.67	1.79	0.004	0.63
FC4	−0.99±0.93	−0.41±0.66	5.1	<0.001*	0.7
FCz	−1.06±1.03	−0.39±0.76	5.21	<0.001*	0.72
T3	−0.003±0.48	0.007±0.53	0.13	0.900	0.02
T4	0.09±0.80	0.02±0.56	0.71	0.482	0.1
T5	0.16±0.81	0.13±0.67	0.3	0.765	0.04
T6	0.29±0.66	0.18±0.69	1.08	0.280	0.16
C3	−0.83±0.87	−0.33±0.69	4.44	<0.001*	0.62
C4	−0.88±0.88	−0.4±0.67	4.32	<0.001*	0.61
Cz	−0.96±1.01	−0.36±0.75	4.8	<0.001*	0.67
TP7	0.22±0.58	0.12±0.64	1.08	0.279	0.16
TP8	0.25±0.64	0.14±0.63	1.25	0.214	0.18
CP3	−0.64±0.80	−0.29±0.69	3.2	0.002	0.46
CP4	−0.68±0.85	−0.29±0.67	3.6	<0.001*	0.51
CPz	−0.75±0.95	−0.31±0.73	3.61	<0.001*	0.51
P3	−0.43±0.78	−0.18±0.67	2.41	0.017	0.35
P4	−0.43±0.81	−0.21±0.65	2.12	0.036	0.31
Pz	−0.5±0.86	−0.22±0.71	2.44	0.016	0.23
A1	0.44±0.48	0.19±0.59	3.04	0.003	0.44
A2	0.49±0.48	0.25±0.58	2.96	0.003	0.43
O1	0.1±0.58	0.08±0.75	0.17	0.865	0.03
O2	0.13±0.63	0.07±0.75	0.62	0.535	0.09
Oz	0.06±0.66	0.07±0.76	0.08	0.934	0.01

* Significant difference between controls and patients with schizophrenia (significance level at 0.0016 after Bonferroni's correction).

Effect size was the standardized mean difference between groups (Cohen's d).

### Prediction model

MMN at frontal electrodes was not correlated with any neuropsychological tests (Table 3) or PANSS scores. Table 4 showed the multiple logistic regression model to predict from which group a subject came. The weighted combination of four factors, MMN amplitude at FCz, block design, arithmetic and performance IQ, could best differentiate the patient group from the control group and fit the observed data well. When using MMN amplitude as the only predictive factor, the percentage of concordant pairs was only 70.0%, and the adjusted generalized R^2^ was only 0.17.

**Table 3:** Correlation between MMN and neuropsychological tests.

	MMN mean amplitude at Fz	
Neuropsychological tests	Correlation coefficient	*P* value
d′	−0.003	0.963
md′	0.023	0.758
Perseverative errors	−0.098	0.188
Conceptual level response	0.101	0.173
Categories achieved	0.067	0.364
Trials to complete first category	0.028	0.703
Arithmatics	0.046	0.545
Digit span	0.023	0.761
Information	0.061	0.430
Digit symbol	−0.079	0.304
Block design	−0.035	0.644
Working memory index	0.082	0.284
Verbal IQ	0.091	0.236
Performance IQ	0.094	0.219
Full IQ	0.059	0.443

Pearson's partial correlation, controlling for age and affected status.

**Table 4:** Multivariate predictive logistic regression model for schizophrenia.

Covariate	Estimate	Standard Error	Wald Chi-square	*P* value	Odds ratio	95% Confidence Interval
Intercept	12.41	2.11	34.64	<0.001	-----	-----
FCz	1.10	0.28	15.20	<0.001	3.01	1.73–5.24
Arithmetic	−0.29	0.09	10.46	0.001	0.75	0.63–0.89
Block design	0.39	0.14	8.04	0.005	1.48	1.13–1.94
Performance IQ	−0.12	0.03	18.56	<0.001	0.88	0.84–0.94

Multiple logistic regression model: *n* = 174, percentage of concordant pairs = 90.5%, percentage of discordant pairs = 9.3%, percentage of tied pairs = 0.1%, adjusted generalized *R* ^2^ = 0.61, Deviance goodness-of-fit test *p* = 0.985>0.05 (df = 169), Pearson goodness-of-fit test *p* = 0.534>0.05 (df = 169), and Hosmer and Lemeshow goodness-of-fit test *p* = 0.816>0.05 (df = 8).

The estimated probability of having schizophrenia (i.e., the *predicted value*,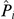) can be calculated using the following formula (FCz is the mean mismatch negativity amplitude at electrode FCz; Arithmetic, Block Design, and Performance IQ are test scores of WAIS-III):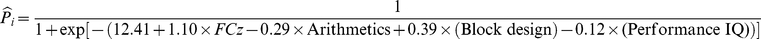.

## Discussion

Our study confirmed MMN deficit of schizophrenia in a Han Chinese population. In concordance with previous studies, duration MMN declined with aging in both schizophrenia patients and controls [27], [40]. Since the baseline MMN amplitude is smaller in schizophrenia patients, the slower declination rate could be due to the “floor effect” [40]. Interestingly, the aging effects on MMN were reported to be related to the length of interstimulus interval. Compared with younger subjects, elder subjects had significantly attenuated MMN when the interstimulus intervals were 4.5 seconds. The aging effects were not evident when interstimulus intervals were only 0.5 seconds [44]. Duration of illness and the chlorpromazine-equivalent dose were uncorrelated with duration MMN after correcting for age.

MMN was not correlated with any of the neuropsychological tests. Several studies have explored the correlation between MMN and neuropsychological tests in small samples of subjects (see Table S1). WCST and CPT were generally uncorrelated with MMN indices, and only Toyomaki et al. reported the duration MMN mean amplitude to be associated with WCST perseverative errors [45]. It is noteworthy that different MMN paradigms and indices yielded different results. For example, Kawabulo et al. found that phonetic duration MMN was correlated with the Rey Auditory Verbal Learning Test, while tone duration MMN was not [46]. Baldeweg et al. found that the MMN memory trace effect, rather than MMN amplitude, was correlated with verbal digit span and pre-morbid verbal intelligence [2].

The weighted combination of MMN and neuropsychological tests enhanced the diagnostic power to differentiate schizophrenia patients from controls. To the best of our knowledge, this is the first study to evaluate the combination of electrophysiological markers and cognitive function for diagnostic purposes. According to previous meta-analyses, the effect sizes of the selected predictors were 1.18 for Arithmetic, 0.46 to 0.84 for Block Design, 1.26 for Performance IQ, and 1.23 for duration MMN [7], [42], [43]. Digit symbol has been reported to have the largest effect size, but it was not selected by the prediction model [42]. There are several reasons why a covariate will be dropped from a prediction model. For example, it may have no effect on the response variable after adjusting for the effects of the other covariates. Further, the correlation of a variable with other covariates can result in collinearity and multicollinearity problems in the regression model.

Our prediction model gave each subject an estimated probability of having schizophrenia, which is unlike the traditional cut-off point method to assign subjects to categorical groups. The probabilistic nature made the prediction model a dimensional assessment, which is emphasized in the proposed Diagnostic and Statistic Manual, fifth edition [47]. The new diagnostic system recognizes cognitive impairment as an important symptom of schizophrenia. However, cognitive impairment is not included as a criteria A symptom due to the lack of diagnostic specificity. For example, Bora et al. argued that the profiles, severity, relationship with clinical states, and prevalence of cognitive impairments do not help to differentiate schizophrenia from other major psychotic disorders. Further, early intellectual declination of cognition exists in a small portion of patients with schizophrenia [48]. Incorporating MMN into the “broad” cognitive assessment could therefore be valuable. MMN impairment is relatively specific to schizophrenia. Abnormal MMN has not been observed in patients with bipolar affective disorder, major depressive disorder or schizoaffective disorder [13], [14]. In addition, MMN and neuropsychological tests are independent and measured by different constructs. A combination of cognitive measures with MMN may thus enhance the differential ability and better address the heterogeneous nature of schizophrenia.

There are several limitations of this study. First, only schizophrenia patients and healthy subjects were compared. The enhanced diagnostic specificity should also be evaluated by comparing schizophrenia to other major psychotic and affective disorders. Second, the subjects were mainly chronic schizophrenia patients, and the results may not be readily applied to first-episode or prodromal subjects. Third, the validity and reliability of the prediction model should be tested in another independent sample. Fourth, all patients in this study were taking psychotropic agents. It could be possible that the prediction model worked by differentiating subjects taking or not taking psychotropic agents, but not subjects with or without schizophrenia. Drug challenge studies in healthy subjects showed that benzodiazepines increased MMN latency [49] and decreased MMN amplitudes [50], and selective serotonin reuptake inhibitors such as escitalopram increased the MMN amplitude [51]. Dopaminergic agents did not influence MMN [52]. In schizophrenia patients, treatment with antipsychotic agents [15]–[19] or benzodiazepines [53], [54] had no effect on MMN, hence the MMN deficit in schizophrenia may not be the result of medications. Further, second-generation antipsychotics have modest effects on improving cognitive function [55]–[57], although first-generation antipsychotics have no or even adverse cognitive effects [57]. Therefore, drug effects seemed to have little influence on the prediction model and the group difference of MMN.

In summary, MMN deficit was a robust phenomenon for Han Chinese schizophrenia patients, and duration MMN decline with increasing age in both schizophrenia patients and healthy subjects. A combination of electrophysiological and neurocognitive markers better differentiated schizophrenia patients from healthy subjects. The multivariate phenotype approach delineated the heterogeneous nature of schizophrenia. As a measurement with good specificity for schizophrenia, future studies should evaluate the value of duration MMN in developing composite diagnostic batteries.

## Materials and Methods

### Subjects

One hundred and twenty stable outpatients, aged 18 to 65 years who met the DSM-IV criteria for schizophrenia, and 76 age- and gender-matched healthy controls were recruited. The study was approved by the Institutional Review Board of the National Taiwan University Hospital. Written informed consent was received from all participants. Each schizophrenia patient's capacity to consent was evaluated by his/her treating psychiatrist who made the referral to this study. When the capacity to consent was reduced, consent from another family member was required in addition to the patient's consent. All subjects were interviewed using the Chinese version of the Diagnostic Interview for Genetic Studies (DIGS) [58]. Then two board-certified psychiatrists independently made the diagnoses according to the DSM-IV-TR criteria by reviewing the DIGS data and medical charts. If the diagnoses were inconsistent, a senior psychiatrist would made the final diagnoses. Subjects with mental retardation, schizoaffective disorders, bipolar affective disorder, organic mental disorders, and substance-related disorders were excluded. The controls had no lifetime or current psychiatric diagnosis or family history of psychotic disorders. Subjects were excluded if they had epileptic disorders or other major brain pathology. The age of onset and Positive And Negative Syndrome Scale (PANSS) scores were recorded for the patients [59]. In addition, daily doses of antipsychotic agents were transformed into chlorpromazine equivalents by the formulas using regression with power transformation by Andreasen [60].

### Electroencephalographic Procedure

The standard protocol for MMN for the experimental paradigm and data processing reported by Light et al. was followed [61]. Audiometry testing was used to exclude subjects who could not detect 40-dB sound pressure level tones at 500, 1000, and 6000 Hz presented to either ear. Subjects were seated in a comfortable recliner in a sound-attenuating and electrically shielded booth. They were instructed to relax and to watch a silent benign cartoon film presented on a 19-inch LCD monitor screen located at eye level to reduce eye movements over the session. During the test session, subjects were closely observed through a video monitor and EEG for signs of sleep or slow wave activity. When encountered, the experimenter spoke briefly to wake up the subject.

The auditory stimuli were generated by a Neuroscan STIM system and were presented to subjects binaurally via foam insert earphones. The data was recorded by a Neuroscan ACQUIRE system (NeuroScan, Inc., El Paso, TX). The EEG signals were recorded with an electrode cap (Quik-Cap, NeuroScan, Inc., Charlotte, NC) from 32 scalp locations (10–20 system). Electrodes placed at the tip of the nose and at Fpz served as the reference and ground, respectively. Four additional electrodes were located above and below the left eye and at the outer canthi of both eyes to monitor blinks and eye movements. Electrode impedances were kept below 5 kΩ prior to MMN recording.

An auditory oddball paradigm of duration MMN of approximately 30-min duration was given. The cartoon soundtrack was turned off and replaced by the experimental 85-dB auditory stimuli, which were presented at a fixed 500 msec onset-to-onset asynchrony. The duration of standard stimulation and deviant stimulation were 50 msec and 100 msec, respectively. Stimuli occurred in a pseudorandom order with probability of occurrence 0.9 for standard tones and 0.1 for deviant tones. Stimuli signals were digitized at a rate of 1 kHz and an on-line band-pass filter at 0.5–100 Hz, without 60-Hz notch filter. During testing, online ERP averages to standard and deviant tones were also acquired to monitor signal quality and the number of sweeps free of gross artifacts (defined as ±100 µV across the −100∼500 msec following stimuli). The MMN session was continued until a minimum of 225 artifact-free deviant trials had been collected on-line.

### EEG data processing

All data were processed using Neuroscan Edit 4.3 software (Compumedics USA, Charlotte, North Carolina). Semi-automated procedures using the Tool Command Language (TCL) batch processing language began with EOG artifact reduction through a built-in pattern-recognition algorithm [62]. The subject's continuous data files were then epoched 100 msec pre-stimulus to 500 msec post-stimulus. Following linear detrending and baseline correction to the average pre-stimulus interval, all epochs containing amplitudes exceeding ±50 µV in frontal recording sites (F7, F8, Fp1, Fp2, F3, F4, and Fz) were automatically rejected. EEG responses to standard and deviant stimuli were separately averaged to create a standard ERP and a deviant ERP, and both were low-pass filtered at 20 Hz (0-phase shift and 24-dB/octave roll-off) to remove any residual high-frequency artifacts. MMN waveforms were generated by subtracting the standard ERP from the deviant ERP [60]. MMN indices were measured as the mean voltage from 135 to 205 milliseconds [63]–[66].

### Neuropsychological Tests

All subjects received MMN examinations, and most of them received the neuropsychological test batteries, including the Continuous Performance Test (CPT) for 114 patients and 70 healthy subjects, Wisconsin Card Sorting Test (WCST) for 115 patients and 71 healthy subjects, and Wechsler Adult Intelligence Scale Third Edition (WAIS-III) for 102 patients and 72 healthy subjects [67]. The detailed procedures of the CPT and WCST have been described in previous publications. In brief, subjects completed two 5-minute CPT sessions: the undegraded 1–9 task, and the 25% degraded 1–9 task [68]. Sensitivity indices indicating the ability to discriminate target from non-target trials were calculated (d′ for undegraded CPT and md′ for degraded CPT). The WCST results were scored as four indices defined in the WCST manual as: (1) perseverative errors: number of errors that were perseverative reflecting the tendency towards perseveration; (2) categories achieved: the number of times 10 consecutive correct responses were made, reflecting overall success; (3) trials to complete first category: number of trials needed to complete the first category; and (4) conceptual level response: proportion of consecutive correct responses occurring in runs of 3 or more, reflecting insight to the correct sorting principles [69]. Index scores of CPT and WCST were transformed to adjusted z score by adjustment for age, sex and education level based on the data of the healthy subjects [68], [70]. The Chinese version of WAIS-III was applied [67]. In addition to Verbal IQ, Performance IQ, and Full Scale IQ scores, several scaled scores of subtests and one composite secondary index were also used, including (1) Information: a measure of acquired general knowledge; (2) Arithmetic: a mental arithmetic task that measures working memory; (3) Digit Span: measuring working memory free from distraction; (4) Block Design: reflection of visuospatial and motor skills; (5) Digit Symbol-coding: assessment of processing speed; (6) Working Memory Index: a composite index composed of Arithmetic and Digit Span.

### Statistical Analysis

Statistical analysis was conducted with the SAS software package, version 9.2 (SAS Institute Inc., Cary, NC, USA). A two-sided p-value less than or equal to 0.05 was regarded to be statistically significant. Continuous data and categorical data were presented with mean±standard deviation (SD) and frequency (percentage), respectively. The Student's t-test and chi-square test were used to compare data between groups. Mixed-model repeated-measures ANOVA was applied for two analyses on the comparison of MMN between patients and controls. The first analysis examined MMN amplitudes recorded from all electrodes over the scalp, with the 32 electrodes as the within-subject factor. The second analysis was focused on midline electrodes, with electrode site (Fz, FCz, Cz, CPz, Pz, and Oz) as the within-subject factor. Group was the only between-subject factor, and age was the only covariate. Huynh-Feldt corrections were applied when the degree of freedom was more than 1. The correlation between MMN at electrode Fz and the neuropsychological tests were explored by Pearson's partial correlation, controlling for covariates significantly associated with MMN.

To identify the predictive factors for schizophrenia, multivariate logistic regression models were applied to find parsimonious regression models that fit the observed data. The MMN value at each electrode and all neuropsychological indices listed were included as potential predictive variables. Then stepwise variable selection procedure was used, where the significance levels for entry and for stay were set to 0.15 initially, and then reduced to 0.05 to identify the best final model. Both the goodness of fit (GOF) measures (the percentage of concordant pairs and adjusted generalized R2) and the GOF tests (deviance GOF test, Pearson chi-squared GOF test, and Hosmer-Lemeshow GOF test) were used to assess the GOF for the fitted model. The percentage of concordant pairs indicated the proportion of “concordant” pairs where case (i.e., the observed binary response is 1) had the highest predicted event probability among all possible case-control pairs. A larger percentage of concordant pairs suggested a better fit of the logistic model. Larger p values were preferred for the three GOF tests when the null hypothesis was that the logistic regression model fit the observed binary data well. Finally, statistical tools for regression diagnostics such as residual analysis, detection of influential cases, and check for multicollinearity were applied [71], [72].

## Supporting Information

Table S1 **Correlation between mismatch negativity and neuropsychological tests.**
(DOC)
