# Supplementary material for: Differentiation of Schizophrenia Patients from Healthy Subjects by Mismatch Negativity and Neuropsychological Tests
Source: PLoS One. 2012 Apr 5;7(4):e34454. doi: 10.1371/journal.pone.0034454 (PMC3320618; doi:10.1371/journal.pone.0034454)
Supplement: Table S1 — Correlation between mismatch negativity and neuropsychological tests. (DOC) [file pone.0034454.s001.doc]

Table S1. Correlation between mismatch negativity and neuropsychological tests

| MMN paradigm | Subjects | Neuropsychological tests with significant correlation | Neuropsychological tests without significant correlation | Reference |
| --- | --- | --- | --- | --- |
| Duration tone MMN  Peak latency  Peak amplitude | Schizophrenia patients  N = 23 | 1. Peak latency: TMT-A 2. Peak amplitude: **WCST-PEN**, Stroop test time, Stroop test error, TMT-A , TMT-B | **WCST-CA**, **WCST-PEM**, **CPT-RT**, **CPT-error**, WFT, TMT time difference | Toyomaki, 2008[1] |
| Duration tone MMN with continuous changing standard stimuli  Mean amplitude  MMN slope | Schizophrenia patients  N = 28  Healthy controls  N = 20 | In patients –  MMN slope: RBMT, **digit span backward and forward** (WMS), MMSE, verbal fluency (COWAT)  In healthy subjects –  Nil. | In patients –   1. Mean amplitude: RBMT, **digit span**, MMSE, verbal fluency, pre-morbid **verbal intelligence** (NART) 2. MMN slope: **verbal intelligence**   In healthy subjects –  All the tests | Baldeweg, 2004[2] |
| Phonetic and tone duration MMN  Mean amplitude | Schizophrenia patients  N = 23 (tone)  N = 14 (phoneme) | 1. Tone duration MMN: nil 2. Phoneme duration MMN: RAVLT | 1. Tone duration MMN: RAVLT, **WCST-CA** 2. Phoneme duration MMN: **WCST-CA** | Kawakubo, 2006[3] |
| Tone duration and frequency MMN  Peak amplitude  Peak latency | Prodromal subjects  N = 43  Schizophrenia patients  N = 31  Healthy controls  N = 33 | No significant findings | In the three groups –  AVMT, verbal fluency, DRT, sustained CPT-d’, **WCST-PE**, MCVT | Brockhaus-Dumke, 2005[4] |
| Duration tone MMN  Mean amplitude | Healthy controls  N = 19 |  | **DSCPT** sensitivity A' (signal/noise discrimination level) | Kasai, 2002[5] |
| Duration tone MMN  Mean amplitude | Schizophrenia patients  N = 19  Healthy subjects  N = 19 | In patients –  D-KEFS Proverb Test, short- and long-delay recall (CVLT-II), GAF  In healthy subjects –  Nil. | In patients –  **WCST-PE**, **WCST-CA**, PPVT, LNS, immediate recall (CVLT-II), UPSA  In healthy subjects  All the tests | Kiang, 2007[6] |
| Duration tone MMN  Mean amplitude  Peak latency | Healthy subjects  N = 20 | Modified GAF (CVLT-II) | WRAT3 reading subtest, **WCST-64-PE**, **WCST-64-CA**, CVLT-II, LNS | Light, 2007[7] |

WCST: Wisconsin Card Sorting Test (-CA: categories achieved; -PE: perseverative error; -PEN: perseverative errors of Nelson; -PEM: perseverative errors of Milner); CPT: Continuous Performance Test (-RT: reaction time; CPT errors: omission and commission errors); WFT: word fluency test; TMT: Trail Making Test; Stroop test; MMSE: mini-mental status examination; RBMT: Rivermead Behavioral Memory Test; WMS: Wechsler Memory Scale; COWAT: Controlled Oral Word Association Test; NART: National Adult Reading Test; RAVLT: Rey Auditory Verbal Learning Test; AVMT: Auditory Verbal Memory Test; DRT: Delayed Response Task; MCVT: Multiple Choice Vocabulary Test; DSCPT: degraded stimuli continuous performance test; D-KEFS: Delis-Kaplan Executive Function System Proverb Test; LNS: Letter-Number Span Test; CVLT-II: California Verbal Learning Test; PPVT: Peabody Picture Vocabulary Test; GAF: Global Assessment of Function; UPSA: UCSD Performance-Based Skills Assessment; WRAT3: Wide Range Achievement Test 3)

Reference

1. Toyomaki A, Kusumi I, Matsuyama T, Kako Y, Ito K, et al. (2008) Tone duration mismatch negativity deficits predict impairment of executive function in schizophrenia. Prog Neuropsychopharmacol Biol Psychiatry 32: 95-99.

2. Baldeweg T, Klugman A, Gruzelier J, Hirsch SR (2004) Mismatch negativity potentials and cognitive impairment in schizophrenia. Schizophr Res 69: 203-217.

3. Kawakubo Y, Kasai K, Kudo N, Rogers MA, Nakagome K, et al. (2006) Phonetic mismatch negativity predicts verbal memory deficits in schizophrenia. Neuroreport 17: 1043-1046.

4. Brockhaus-Dumke A, Tendolkar I, Pukrop R, Schultze-Lutter F, Klosterkotter J, et al. (2005) Impaired mismatch negativity generation in prodromal subjects and patients with schizophrenia. Schizophr Res 73: 297-310.

5. Kasai K, Nakagome K, Hiramatsu K, Fukuda M, Honda M, et al. (2002) Psychophysiological index during auditory selective attention correlates with visual continuous performance test sensitivity in normal adults. Int J Psychophysiol 45: 211-225.

6. Kiang M, Light GA, Prugh J, Coulson S, Braff DL, et al. (2007) Cognitive, neurophysiological, and functional correlates of proverb interpretation abnormalities in schizophrenia. J Int Neuropsychol Soc 13: 653-663.

7. Light GA, Swerdlow NR, Braff DL (2007) Preattentive sensory processing as indexed by the MMN and P3a brain responses is associated with cognitive and psychosocial functioning in healthy adults. J Cogn Neurosci 19: 1624-1632.
